# Deleterious effect of angiotensin-converting enzyme gene polymorphism in vitiligo patients

**DOI:** 10.1016/j.sjbs.2021.04.045

**Published:** 2021-04-24

**Authors:** Nosiba Suliman Basher, Abdul Malik, Fahad Aldakheel, Anis Ahmad Chaudhary, Hassan Ahmad Rudayni, Musaed Alkholief, Aws Alshamsan

**Affiliations:** a Department of Biology, College of Science, Imam Mohammad Ibn Saud Islamic University, Riyadh, Saudi Arabia; b Nanobiotechnology Unit, Department of Pharmaceutics, College of Pharmacy, King Saud University, Riyadh, Saudi Arabia; c Department of Pharmaceutics, College of Pharmacy, King Saud University, Riyadh, Saudi Arabia; d Department of Clinical Laboratory Sciences, College of Applied Medical Sciences, King Saud University, Riyadh, 11564, Saudi Arabia; e Prince Sattam Chair for Epidemiology and Public Health Research, College of Medicine, King Saud University, Riyadh, Saudi Arabia

**Keywords:** *ACE* gene, Insertion, Deletion, Polymorphism and Vitiligo

## Abstract

Vitiligo is a rare skin condition caused by an immune reaction. Vitiligo can occur anywhere on the body. This proposed explanation of vitiligo makes it clear that vitiligo is not linked to any other autoimmune diseases. The polymorphisms of some genes present in the immune system play a major function in susceptibility of vitiligo. Meta-analysis studies have shown that the Angiotensin converting enzyme (ACE) gene insertion and deletion polymorphism is closely associated with vitiligo in many ethnicities. The connection between *ACE* gene and vitiligo is connected through the auto immune diseases and there are no genetic polymorphism studies have been carried out with ACE gene with vitiligo in the Saudi population. Previous studies show that vitiligo patients are more likely to also have an autoimmune disorder. The current study aims to investigate the I/D polymorphism in the ACE gene with diagnosed patients with vitiligo subjects. This is a case-control study carried out in the Saudi population with 100 vitiligo cases and 100 healthy controls. Genotyping was performed through polymerase chain reaction followed by 3% agarose gel electrophoresis. Genotype and allele frequencies were carried out with genetic mode of inheritances. Statistical analysis was performed considering p < 0.05 as significant association. There was a substantial difference in allele frequency distribution between vitiligo patients and healthy controls (OR-1.70 (95%CI: 1.14–2.53); p = 0.008). Additionally, DD genotype (OR-4.71 (95%CI: 1.42–15.61); p = 0.008) and recessive model (OR-2.66 (95%CI: 1.41–5.02); p = 0.002) was strongly associated. Both dominant and co-dominant showed the negative association (p > 0.05) when compared between the vitiligo cases and controls. The correlation between age and genotyping was performed with Anova analysis and current study results confirmed the substantial link between 11 and 20 years (p = 0.01) and 31–40 years (p = 0.04) with the defined age groups. In conclusion, in Saudi populations, the *ACE* gene I/D polymorphism was identified as being correlated with vitiligo. This is the first study in Saudi Arabia to report the risk factors of vitiligo with the *ACE* gene polymorphism.

## 1 Introduction

Vitiligo is a widespread chronic depigmentation of the skin, caused by the selective disruption of the functioning of melanocytes in the skin, hair or both (Ahmed et al., 2020). Vitiligo is a severe, acquired, idiopathic and progressive skin disease characterized by the loss of functional melanocytes and melanin from hair follicles and skin (Almohideb, 2020). The disease vitiligo exists in a small fraction (0.5–10%) of the world population. Most people develop vitiligo before they have reached the age of 40. The causes of vitiligo are still unclear (Cheng et al., 2020). The loss of melanocytes and dark pigment development of melanin is the result of medical conditions linked to the immune system and our environment (Rajendiran et al., 2020). The precise cause of vitiligo has yet to be determined (Yan et al., 2020). Vitiligo is largely dependent on genetics and is a multifactorial disorder, meaning it has many genetic causes. Vitiligo is a polygenic disorder and many genes related to autoimmunity are associated with its pathogenesis (Huraib et al., 2020).

Several genetic factors were identified that control the role of the immune system. One essential gene is Angiotensin converting enzyme (ACE), which controls the vasculature and also participate in the immune system. The D allele of the *ACE* gene insertion/deletion (I/D) polymorphism was found to be linked to vitiligo (Patwardhan et al., 2013, Rashed et al., 2015). Angiotensin II is considered an essential regulator of the renin-angiotensin system (RAS) and kallikrein-kininogen systems. ACE has a major function in the body's inflammation phase and it has been studied in conjunction with several autoimmune diseases (Rashed et al., 2015). The *ACE* gene contains an intron of an intron of 287-bp DNA (ALU sequence) absorbent polymorphism dependent on presence (I-insertion) and absence (D- removal), resulting in three genotypes (homozygous II and DD, and ID heterozygotes). ACE, the renin-angiotensin system, is an essential enzyme encoded with the ACE gene on chromosome 17q23 consisting of 26 exons and 25 introns (Alharbi et al., 2013a, Alharbi et al., 2013b). The D allele was shown to be dose-dependently correlated with higher circulatory ACE enzyme (Poornima et al., 2015).

Several studies have explored the connection between these polymorphisms and autoimmune disorders (Hassan et al., 2019, van Belle et al., 2011). Numerous studies have shown the relationship between *ACE* gene I/D polymorphism and vitiligo. Many genetic findings on the interaction of *ACE* I/D polymorphism with and without vitiligo are inconclusive (Almohideb, 2020, Patwardhan et al., 2013, Rashed et al., 2015). The current study aims to explain the relation between of ACE gene I/D polymorphism with vitiligo.

## 2 Materials and methods

### 2.1 IRB approval

The initial step for this study was approving the ethical grant from Institutional Review Board at King Saud University (KSU). The patients have signed the informed form before enrolling in this study based on inclusion and exclusion criteria of vitiligo cases and control subjects

### 2.2 Patient recruitment

This is a prospective case-control study carried on in KSU premises. The vitiligo patients were recruited from outpatient clinic at Dermatology department in KSU, Riyadh and Saudi Arabia. In this case-control study, we have recruited 100 vitiligo patients and 100 controls without age matching and gender was collected equally. The inclusion criteria of the vitiligo cases were adopted from based on Vitiligo European Task Force (Taïeb et al., 2007). The exclusion criteria of the vitiligo cases are based on other skin diseases such as eczema (Bogari et al., 2020) apart from vitiligo. The inclusion criteria of the control subjects are patients confirmed without any skin and other diseases. The exclusion criteria of the controls were diagnosed with any health issues or diseases.

### 2.3 Anthropometric details

In this study, we have recorded the anthropometric details such as age, gender, family history and specialized category of the vitiligo disease which were tabulated in Table 1.

**Table 1:** Anthropometric characteristics between Vitiligo cases and controls.

	**Vitiligo patients** **(n = 100)**	**Control** **(n = 100)**	**p-value**
Gender Male Female	45 (45%) 55 (55%)	45 (45%) 55 (55%)	1.00 1.00
Age (years)	(27.1 ± 13.22)	(41.04 ± 17.06)	0.88
Disease Duration (years)	5.0 ± 2.1	NA	NA
Generalized Clinical type of Disease	100 (100%)	NA	NA
Family history	31 (31%)	NA	NA

### 2.4 Blood and genetic analysis

In this study, 2 ml of whole blood was collected in an EDTA vacutainer and DNA was extracted using the genomic DNA isolation kit (Qiagen, USA). NanoDrop spectrophotometer was used to measure the DNA quantity. Samples were stored at freezer for further usage.

Genotyping was performed by direct polymerase chain reaction (PCR) technique to study the Alu287bp sequence of insertion and deletion polymorphism in the *ACE* gene using Thermo Scientific DreamTaq Green PCR master mix (Ig BioSystems, Saudi Arabia). The PCR was performed with 50 μl reaction which consists of 25 μl of master mix which consists of 10X buffer, MgCl_2_, dNTPs and Taq DNA polymerase. Apart from this we have added 10 μl of double distilled, 2 μl of forward and reverse primers of 10pmoles. Genomic DNA of 5 μl was added separately into each tube. The primer sequences were grabbed from (Khan et al., 2014, Alshammary and Khan, 2021) studies of forward sequence and reverse sequence as follows

F-5′CTGGAGACCACTCCCATCCTTTCT3′.

R-5′GATGTGGCCATCACATTCGTCAGAT3′

**Fig. 1:**
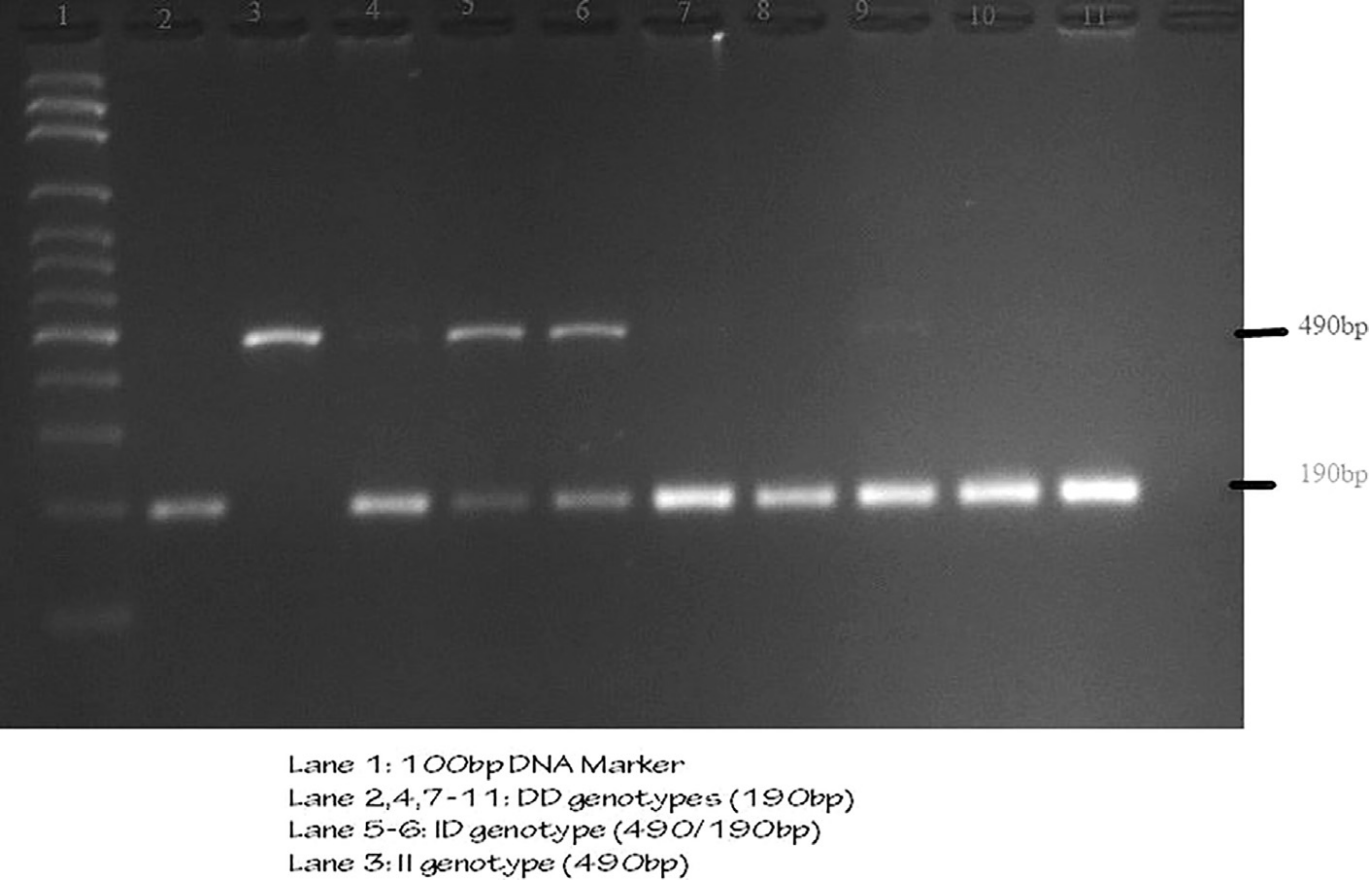
Ethidium bromide stained 3% agarose gel electrophoresis for *ACE* gene.

PCR was performed by initial denaturation at 95C for 10 min followed with denaturation at 95C for 30 s. Annealing is the most important and second step which is carried out 58C for 30 s and in the final step of extension was carried out at 72C for 45 s. The final denaturation was 72C for 10 min. The overall cycle was carried out at 35 rounds and holds finally at 4C. Agarose gel of 3% was prepared with 3 g of agarose in 100 ml of 1X TBE buffer and mixed with 10 μl of ethidium bromide. The PCR products was run to analyse the *ACE* I/D polymorphisms on agarose gel and visualized under UV transilluminator. The band sizes for II genotype was 490 bp and DD was 190 bp. The heterozygous ID was 490/190 bp respectively (Fig. 1).

### 2.5 DNA validation

In this study, we have selected randomly 15% of samples from cases and controls to validate the obtained results in our study. The complete Sanger sequencing was performed as per the described methodology (Alharbi et al., 2021).

### 2.6 Statistical analysis

In this study, SPSS software version 25.0 was used to perform the statistics between the vitiligo cases and controls. Mean and standard deviation was represented as continuous variables and the qualitative was represented in frequencies and percentages (Table 1). Hardy Weinberg equilibrium (HWE) was performed with one-degree of freedom (Table 2). Statistical analysis between the vitiligo cases and controls using genotyping data with odds ratios and 95% confidence intervals was performed with both genotype and allele frequencies including dominant, co-dominant and recessive mode of inheritances (Table 3). One-way Anova analysis was performed between the correlation of age and genotypes. All the Pvalues were considered as < 0.05 as significant (See Table 4.)

**Table 2:** Hardy Weinberg Equilibrium in *ACE* gene Insertion and Deletion polymorphism studies.

**Gene**	**Sequence**	**Control genotypes(II/ID/DD)**	**χ^2^**	**P value**
***ACE***	Alu deletion 287 bp	10/80/80	36	p < 0.00001

**Table 3:** Genotype and allele frequencies between Vitiligo cases and non-vitiligo subject.

**Various mode of inheritances**	**Vitiligo (n = 100)**	**Controls (n = 100)**	**ORs**	**95% CI**	**p Value**
**II (Insertion)**	07 (7%)	10 (10%)	Reference	Reference	Reference
**ID (Insertion/Deletion)**	60 (60%)	80 (80%)	1.07	0.38–2.97	0.89
**DD (Deletion)**	33 (33%)	10 (10%)	4.71	1.42–15.61	0.008
**Dominant Model**	93 (93%)	90 (90%)	1.47	0.53–4.04	0.44
**Co-dominant Model**	60 (60%)	80 (80%)	0.37	0.19–0.70	0.002
**Recessive Model**	40 (40%)	20 (20%)	2.66	1.41–5.02	0.002
**I allele**	74 (0.37)	100 (0.50)	Reference	Reference	Reference
**D allele**	126 (0.63)	100 (0.50)	1.70	1.14–2.53	0.008

**Table 4:** Correlation between age and genotype frequencies.

**n (%)**	**Age range**	**II (n = 7)**	**ID (n = 60)**	**DD (n = 33)**	**P value**
**n = 03**	1–10	01 (14.3%)	01 (1.7%)	01 (3%)	1.00
**n = 36**	11–20	03 (42.8%)	21 (35%)	12 (36.4%)	0.01
**n = 27**	21–30	01 (14.3%)	15 (25%)	11 (33.3%)	0.06
**n = 20**	31–40	02 (28.6%)	13 (21.7%)	05 (15.2%)	0.04
**n = 09**	41–50	00 (0%)	06 (10%)	03 (9.1%)	0.08
**n = 03**	51–60	00 (0%)	02 (3.3%)	01 (3%)	0.68
**n = 02**	61–70	00 (0%)	02 (3.3%)	00 (0%)	0.06

## 3 Results

### 3.1 Descriptive characteristics

The descriptive characteristics of vitiligo cases and controls have been documented in Table 1. Based on the anthropometric data, the mean age of the vitiligo cases was found to be 27.1 ± 13.22 and 41.04 ± 17.06 was documented in the control subjects. In this study, 55% of females and 45% of males were equally selected in both the vitiligo cases and controls. The mean age of the vitiligo disease was documented to be 5.0 ± 2.1. The family history was documented to be 31% in the vitiligo cases. However, there was no data in controls for any family history of vitiligo and mean age. The *t*-test for age (p = 0.88) and gender (p = 1.00) was non-significantly associated when compared between vitiligo disease and control subjects (p > 0.05).

### 3.2 HWE equilibrium

The genotype distribution between ACE gene I/D polymorphism were not in the agreement with those predicted through the HWE in the control group (χ^2^ = 36 and p < 0.00001). The details were documented in Table 2.

### 3.3 Genotyping analysis

**Fig. 2:**
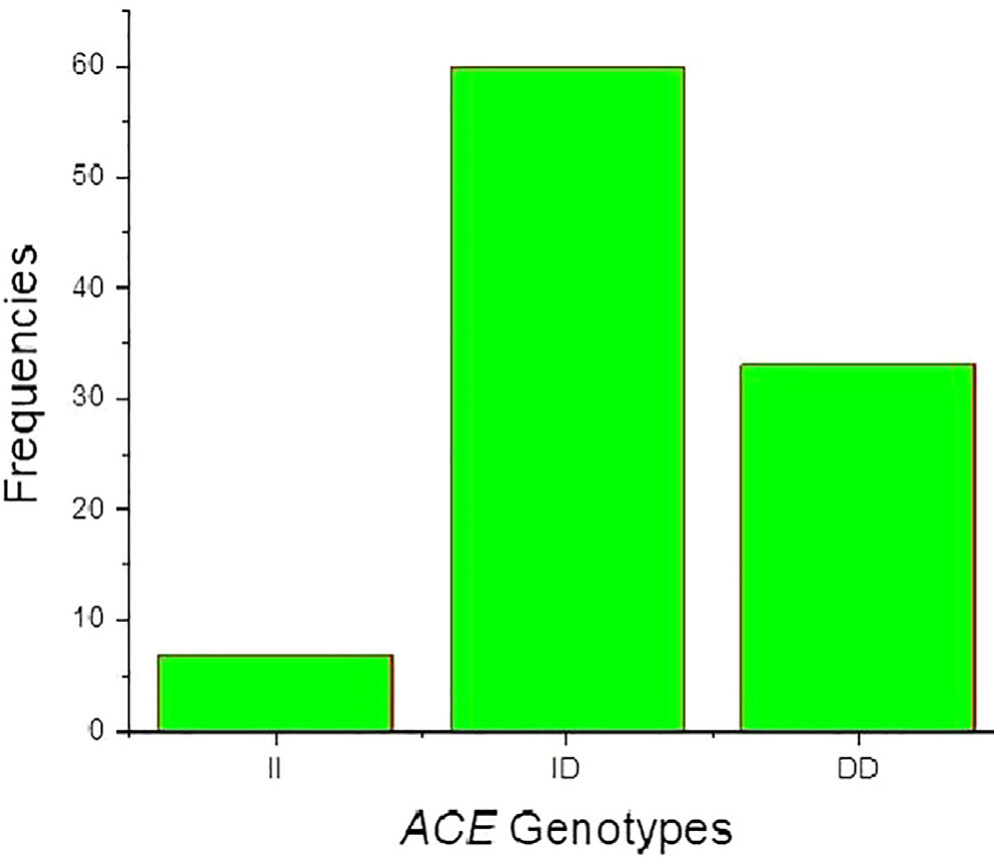
Representation of genotype frequencies in *ACE* gene.

**Fig. 3:**
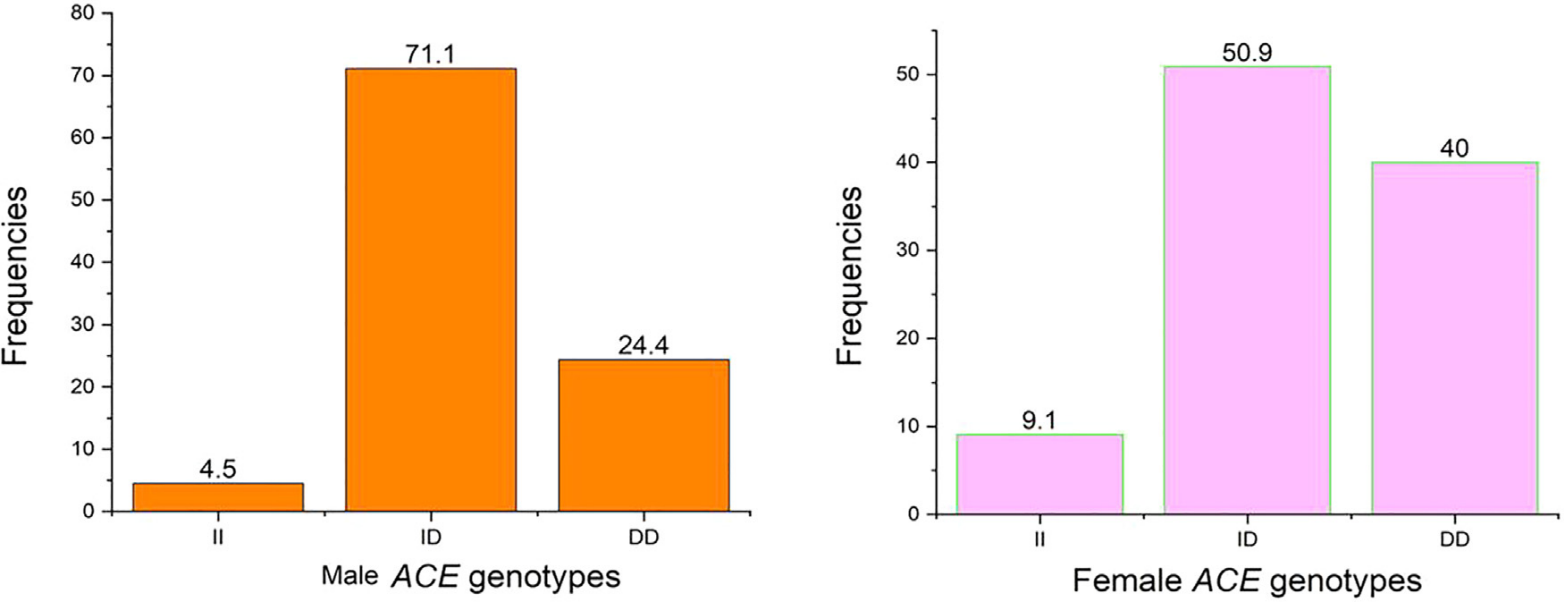
Gender based representation of *ACE* genotype frequencies in vitiligo cases.

The I and D alleles and the II, ID and DD genotypes at position rs4646994 were identified in cases and controls of the *ACE* gene. Allele and genotyping frequencies of *ACE* gene I/D polymorphism in vitiligo cases and control subjects was shown in Table 3. The I allele frequency was 37% and D allele frequency was 63% in vitiligo cases and in controls both the I and D allele frequencies was documented to be 50% of each. The genotype distribution of ACE gene I/D polymorphism II, ID and DD genotypes in cases was 7%, 60% and 33%, whereas in controls the II, ID and DD genotypes was 10%, 80% and 10% respectively. The DD genotype (DD vs II: OR-4.71 (95%CI: 1.42–15.61); p = 0.008), D allele (D vs I: OR-1.70 (95%CI: 1.14–2.53); p = 0.008) and recessive models (OR-2.66 (95%CI: 1.41–5.02); p = 0.002) was strongly associated when compared between the vitiligo cases and controls. Both the dominant (OR-1.47 (95%CI: 0.53–4.04); p = 0.44) and co-dominant (OR-0.37 (95%CI: 0.19–0.70); p = 0.002) models didn’t showed any association in this study. The Fig. 2 represents the genotype frequencies in vitiligo cases and Fig. 3 represent the genotype frequencies in vitiligo cases of both male and female participants.

### 3.4 Correlation between age and genotyping

In this study, we have categorized age into 7 categories as 0–10 (n = 03), 11–20 (n = 36), 21–30 (n = 27), 31–40 (n = 20), 41–50 (n = 09), 51–60 (n = 03) and 61–70 (n = 02) respectively. The II, ID and DD genotypes was found to be high in the age category between 11 and 20 years (II = 03, ID = 21 and DD = 12). The one-way Anova analysis was found to be significantly associated with the age categories between 11 and 20 years (p = 0.01) and 31–40 years (p = 0.04).

## 4 Discussion

Vitiligo is a confounding genetic disorder that may include genes expressed in cells of the skin (Jin et al., 2004). Another important hypothesis for vitiligo pathogenesis, particularly for sectoral vitiligo, is an abnormality affecting the neural system. In the presence of noxious stimuli such as chemical and mechanical injuries, neuropeptides such as skin P substance may be released from sensory nerves, then trigger or increase inflammatory reactions including plasma extravasation, leukocyte activation and cytokine development and mast cell activation. Angiotensin-converting enzymes can inactivate bradykinin, modulate skin-neurogenic inflammation and P- and another neuropeptide degradation (Zhang et al., 2005). The purpose of this study was to study *ACE* gene I/D polymorphism gene association in vitiligo cases. This research is confirmed by the fact that the initial study carried out in the Saudi population confirmed the substantial correlation with our current findings. The findings of our study show that D allele, DD genotype and recessive mode of heritages are associated to the *ACE* gene polymorphism. Alu-287 bp can therefore be identified as a risk marker based on global study. ACE catalyze the production of potent angiotensin II vasoconstrictor by angiotensin I. It is an enzyme that controls body fluid volume, fluid and electrolyte concentrations and regulates human blood pressure in the renin-angiotensin-aldosterone system (RAAS). In addition, ACE position is important for RAAS (Sabir et al., 2019).

There is limited evidence that ACE may require the development of vitiligo. The SP (substance P) and other peptide mediators can be degraded by ACE. Neuropeptides such as SP, which respond with harmful stimuli such as chemical and mechanical injury, are released from the sense nerves in skin. Inflammatory responses such as plasma extravasation, activation of leucocytes, development of cytokines and mast cell activation may be triggered or enhanced by SP. Another potential mechanism is that melanocytes may vary from patient to monitor for *ACE* gene expression.

ACE refers to the transfer of angiotensin into its active form, angiotensin II. The ACE gene is located on the chromosome 17 long arm (17q23.3). The gene consists of 26 exons and 25 introns. As of 2012, scientists have established about 160 ACE gene variants. In the coding area, there were only 34 polymorphisms. The first to report the polymorphism of insertion/deletion (I/D) from ACE was Rigat and colleagues (Rigat et al., 1992). The inclusion (insertion) or absence (deletion) of an AluYa5 factor in intron 16 of this variety resulting in two genotypes (II homozygote, ID heterozygote and DD homozygote). While, I/D polymorphism is in a non-coding area of the ACE genome, the D allele has shown to contribute to an increased circulating serum activity of ACE, indicating the presence of this mutation in ACE levels. In this DD genotype the maximum serum ACE activation was observed while in II genotype the lowest was identified (Khan et al., 2014, Sameer et al., 2010, Poornima et al., 2015).

Previous global studies have shown an important correlation with *ACE* gene polymorphism in vitiligo studies (Pradesh, 2011, Jin et al., 2004, Almohideb, 2020, Rashed et al., 2015, Azeem et al., 2016, Akhtar et al., 2005, Deeba et al., 2009). However, other global studies have shown a negative correlation with *ACE* gene polymorphism in Vitiligo disease (Akhtar et al., 2005, Azeem et al., 2016, Dwivedi et al., 2008, Pradesh, 2011). Recent meta-analysis studies were performed with a large sample size in vitiligo disease with *ACE* gene polymorphism and found a negative correlation (Almohideb, 2020). However, (Patwardhan et al., 2013) case-control study and meta-analysis was documented to be associated significantly. Our current results have been consistent with previous associated studies in vitiligo with *ACE* gene polymorphism. The DD genotype is similar in our sample with a prevalence of 33% to the previous Rashed et al study (Rashed et al., 2015).

The *ACE* gene has been used for I/D polymorphism, with several diseases in the Saudi population and both positive and negative associations are confirmed (Al-Harbi et al., 2015, Alharbi et al., 2013a, Alharbi et al., 2013b, Al-Saikhan et al., 2017, Settin et al., 2009). The frequencies of the genotype differed depending on the human diseases and their frequencies. There are some limitations to the current study. This study is initially constrained by a single polymorphism. ACE serum levels were lacking the secondary limitation of this analysis. The final limitation was not that the expression experiments were carried out. Our study has several strengths, including the recruitment of Saudi subjects in hospital premises, the significant *ACE* genetic polymorphism in vitiligo and control subjects, and the confirmation of the findings of the study currently validated by sanger sequencing. In conclusion, our findings demonstrate that *ACE* I/D polymorphism could serve in the vitiligo as a possible genetic modulator. In particular, our data show that *ACE*, DD genotypes are vulnerable to the risk of developing vitiligo in vitiligo patients. In other Arab countries a similar study should be done to exclude it. Future vitiligo studies can concentrate on multiple RAAS genetic markers of large sample size.

## Declaration of Competing Interest

The authors declare that they have no known competing financial interests or personal relationships that could have appeared to influence the work reported in this paper.
